# Cinnamon-mediated nano emulsion and its cryogenic activity on ROS-affected human sperm—A short study

**DOI:** 10.1016/j.toxrep.2024.101714

**Published:** 2024-08-20

**Authors:** Parameswari Ranganathan, TB Sridharan, R. Babujanarthanam, P. Madhan Kumar, R. Ganesamoorthy, K. Thirugnanasambandham

**Affiliations:** a Department of Biotechnology, Thiruvalluvar University, Serkkadu, Vellore 632115, India; b SMV 117, Gene Cloning and Technology lab, SBST, VIT, Vellore, India; c Department of Chemistry, Vinayaka Mission’s Kirupananda Variyar Arts and Science College, Vinayaka Mission’s Research Foundation (DU), Salem, Tamil Nadu 636308, India; d Tamil Nadu Agricultural University, Lawley Road, Coimbatore, Tamilnadu 641003, India

**Keywords:** Cryo-nano emulsion, Reactive oxygen species, Vitality, Acrosomal layer, Morphology, Cinnamon

## Abstract

Oil-mediated nano emulsion, a more advanced technology than other commercial extenders, will protect spermatozoa for extended periods against ROS and cryo injuries. The study is designed to elucidate the most suitable extender to preserve infertile subjects' sperm cells against ROS damage. This is a significant step forward for the andrology society, as it introduces the use of a nano emulsion extender (natural oil extract) for the first time, particularly for those who are undergoing urology-related defects. The study involved forty-four (44) infertile subjects with smoking habits and forty-two (42) fertile subjects without smoking habits, as per CASA guidelines. Semen preservatives (glycerol and egg yolk citrate), along with our naturally derived nano emulsion components (CN, EN, and EL), were prepared and used to preserve the semen sample for 24 hours. Standard semen parameters (morphology, viability, and integrity), ROS, and sperm acrosome state by staining were measured before and after cryopreservation. The results indicated that the morphology and motility of sperm features were better maintained in the CN-oil-mediated nano emulsion than in other emulsions, and ROS-induced DNA damage was reduced.

## Highlights

• Cryopreservatives generally used to protect sperm cells during the person who undergone surgery or donated sperm at sperm bank for future purposes.
• Nano emulsion is a new trend in sperm cryopresrvation. Nanoemulsion from natural sources than commercial will be less/zero side effects.
• Less ROS or sustained stability of sperm cells were observed under nanoemulsion than other meidum. Easy preparation than Nano particles & cost.

## Graphical Abstract

**Figure:**
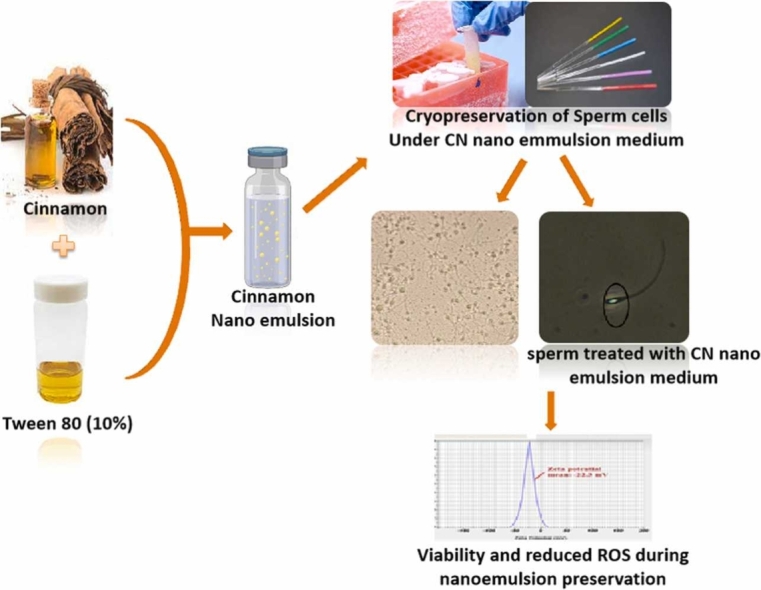
Graphical Abstract

## 1 Introduction

Infertility is the failure to conceive a pregnancy after one year without unprotected sexual intercourse [1]. Male infertility is a man's inability to conceive a child with a fertile female. Pregnancy has never occurred in cases of primary infertility. After a year of trying, one of the partners in a couple cannot achieve egg-sperm oocyte fusion. It is mainly owing to a shortage of semen in the case of a male, and semen quality is the primary indicator of male output. It is responsible for 41–51 % of infertility in humans. It affects approximately 7 % of all men. [2]. This decline could be caused by habitat, occupation, and an improved lifestyle. Men undergoing therapy must, therefore, maintain their sperm cells for future use [3]. Sperm cryopreservation has been used to treat infertile couples since the 1970s. According to studies, several preservatives are available in andrology and sperm banks. As a result, few are permitted to use them, and they fail to sustain sperm motility and viability. The current work is based on examining a sperm sample that was cryopreserved using nano emulsions. Recently, the nano-based cryo-preservation approach has been used. It has been used to preserve dormant but potentially alive living cells and tissues for cryopreservation. Nano emulsions are emulsions with nanosized droplets designed to enhance the delivery of active medicinal components [4]. This is the first attempt at creating a cryopreservation nano emulsion. Nano emulsions consist of tiny droplets with a larger surface area, allowing for improved absorption. They are available in various formulations, such as foams, creams, liquids, and sprays. In cell culture technology, nano emulsions enhance the uptake of oil-soluble nutrients and aid in the solubilization of lipophilic medicines [5]. Nano emulsions are a recent trend with various applications, including cancer treatment. These nano emulsions are also eco-friendly and cost-effective. A previous study found that zinc oxide nanoparticles had a positive effect on sperm shape and preservation compared to other preservatives [6]. Consequently, this short study concentrates on different types of nano emulsions and their protective impact on human sperm cells.

## 2 Materials and methods

### 2.1 Ethics

This study was approved by the institutional review board of VIT University, with Ref. No. VIT/UHEC-3/NO.11; informed consent form was attained and kept privately.

### 2.2 Exclusion criteria

Patients having a history of genital checking, sexually transmitted infections, genital trauma, testicular varicocele, genital infection, family inheritance, toxins, systemic and chronic disorders, medicines influencing lipid metabolism, chemotherapy, and radiotherapy were excluded from the study. This study eliminated patients above 40, those with STDs, and those with occupational inheritance (pressure, thermal energy). Hormonal abnormalities, autoimmune illnesses such as hepatitis B and type 1 diabetic patients, and those with sedentary lifestyles were excluded from this study.

### 2.3 Inclusion criteria

Patients in the current study have smoked for at least 6–8 years. The study also included patients with habits such as drug misuse, inactive lifestyles, and regular junk food eaters. The study only targeted male infertility spouses with fertile female partners (aged 21–39 years).

### 2.4 Collection of semen sample

Semen is collected from the Bangalore Assisted Conception Center- Milann Fertility centre (BACC-Milann), Bangalore, Karnataka, India. Sterile containers were used to collect sperm cells, and the sperm were analysed according to World Health Organization guidelines [7]. The study subjects were divided into fertile (N = 44) and infertile (N = 44) groups based on the significant conventional semen analysis report by computer-assisted semen analysis (CASA), stem motility (fast and slow), morphology, and count. The semen samples were transported to the Vellore Institute of Technology using a liquid nitrogen(-196ºC) transporter for further nano emulsion and stability validation analysis.

### 2.5 Preparation of nano emulsions

#### 2.5.1 Collection of nano emulsions

The three different Nanoemulsions, namely Cinnamon (C1), Clove (C2), and Eugenol (E) oils, were purchased and collected in sterile tubes.

#### 2.5.2 Cinnamon oil (CN)

The surfactant Tween 80 (10 %) was titrated into an aqueous phase with cinnamon oil and coconut oil at a ratio of (2:8 – 10:0) [8].

#### 2.5.3 Clove oil (CE)

The surfactant Tween 80 (0.5 %) was titrated into an aqueous phase with either 75 % corn oil or 50 % medium-chain triacylglycerol (MCT) into clove oil before homogenization [9].

#### 2.5.4 Eugenol oil (EL)

The surfactant Tween 20 was titrated into an aqueous phase and added gently with Eugenol (C10H12O2), a phenyl propanoid [10].

### 2.6 Sperm membrane protein estimation, vitality, and viability check

The sample's total sperm membrane protein content was estimated by the Lowry et al. [11] method. To examine the Vitality Health Check of sperm, we adopted a protocol [12]. Sperm stability is the percentage of viable sperm in a sperm sample. The test was performed according to Garolla. et al. [13]**.**

### 2.7 Reactive oxygen species and acrosomal staining using NBT dye

To analyse the ROS in seminal plasma, we followed the protocol using Nitro Blue Tetrazolium (NBT) dye, and standardized in our laboratory (with few modifications to the regularly used method) [14]. This was followed by analysing the acrosomal layer in the semen sample using a few modifications, according to Agarwal et al. [12].

### 2.8 Statistics

All the statistical analyses were done with Graph Pad Prism version 6.0. All the results were represented as the mean ± standard error of the mean (SEM). Significance differences with subjects are expressed with P<0.001.

## 3 Results

Based on CASA results, the subjects were categorized as fertile and infertile subjects. However, the major semen parameters from fertile (control) and infertile subjects are listed in Table 1. Then, the sperm cells split into five aliquots at five different intervals. Then, it was allowed to be treated with three types of nano emulsion and incubated at room temperature based on the time intervals. The sperm cells were then checked. Based on the number of viable cells and their viable morphology basis, Cinnamon Oil (CN) was adapted to the sperm cell character. It had fewer damages than other nano emulsions, as mentioned in Table 2. Hence, further analysis was carried out with CN Nano Nanoemulsioned on the viability of sperm cells, and a preservative of CN Nano medium was again used to check sperm count, sperm morphology, and other sperm velocity characters. The results in Table 1 explain that the high ROS levels were negatively correlated with morphology and rapid progressive motility of infertile semen samples. CN-treated sperm cells showed rapid motility (1.68±0.47***) and normal morphology (2.0±0.5***) with infertile subjects' near-to-fresh rapid motility and morphology values. ROS and sperm morphology were statistically significant with *P*<0.001, & *P*<0.05 of one-way analysis of variance (ANOVA).

**Table 1:** Semen parameters, biochemical and oxidative damage analysis and its comparison between fertile and infertile subjects of fresh and nano-emulsion preserved (post-thawed) sperm cells and its values.

Semen parameters	Before Preservation		After Preservation	
	Fertile (N=42)	Infertile (N=42)	Fertile (N=42)	Infertile (N=42)
Sperm count (millions/ml)	47.15 ± 7.46	15.69 ±3.90	43.12± 1.34	11.98±2.11
Total Motility (%)	40.50 ±4.53	15. 23 ± 3.20	37.21±0.94	9.56±0.43
Rapid Progressive Motility (%)	21.10 ±3.20	2.00 ±0.52	18.18±2.86	1.68±0.47***
Morphology (%)	17.20 ±1.70	2.5 ± 0.46	15.74±1.21	2.0±0.5***
Head Defects (%)	0.15±0.0005	21.556±0.230	10.02±0.04	23.81±0.697
Tail Defects (%)	0.32±0.0004	19.34±2.14	3.05±0.09	12.93±0.184
Sperm membrane proteins (mg/ml)	21.42±2.63	15.24±0.93	19.88±0.82	10.94±0.85***
Sperm ROS (scores)	3.21±0.04	24.1±0.006	3.81±0.28	28.47±2.06***

Values are represented here Mean± standard error of the mean (SEM), ***: negative correlation with *P*<0.001 significant.

**Table 2:** explains that Nano-emulsions effects on sperm viability were shown in selected time intervals). The Presence of Live Sperms after Addition of Oil at certain Time Intervals. Temperature = 37°C.

Time Interval	2hrs	4hrs	6hrs	12hrs	24hrs
Cinnamon Oil (EN)	100	98	93	91	89
Clove Oil (CE)	100	97	92	89	85
Eugenol Oil (EL)	100	95	91	88	83

### 3.1 Microscopic examinations of CN treated nanoemulsion medium and its effects on infertile sperm cell

**Fig. 1:**
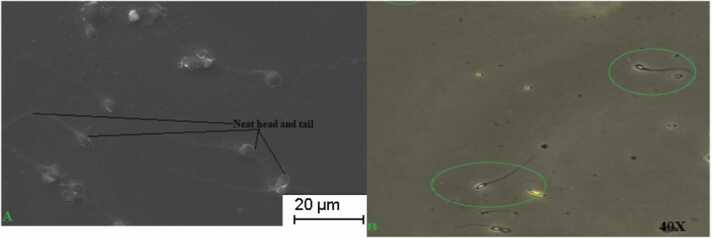
The one full sperm is identified from Fertile smokers and infertile smokers sample after CN cryo-preservation and its microscopic examination for viability. CN medium treated sperm cells under Scanning Electron microsope shows clear outer surface, head, tail and neat acrosome confirms sperm cells were sustained from further ROS damage. Infertile sperm cells preserved under CN- nanoemulsion and its viability under 40X light microscope.

**Fig. 2:**
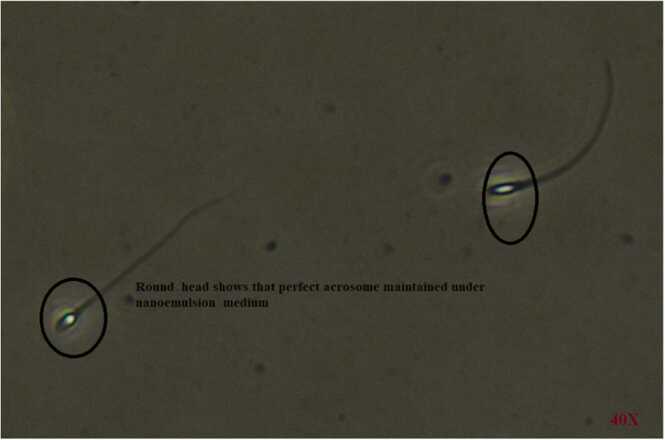
The perfect acrosome layer was highlighted in the round under CN treated Nanoemulsion medium.

CN medium-treated sperm morphology was given in Fig. 1 of A and B. Here, we have given head and neck mid-piece defects, and the tail of sperm was maintained as well as sustained from further ROS damage. Hence, the CN medium proved its ability to maintain sperm morphology, making it more fertile than infertile. Also, it proves and maintains the sperm characteristics, which sustains the environmental damage of ROS. Fig. 2 shows the acrosomal part of sperm treated with CN medium. Like sperm morphology and count parameters, the acrosomal part of sperm was obtained without any damage, as viewed in Fig. 2. So, from the overall results, based on the viability check, we have selected CN for further semen parameter analysis.

## 4 Discussion

We recently reported that human sperm cryopreservation is also possible. Regarding reproductive concerns, those recently recovered from SARS-CoV-2-affected COVID-19 patients had mineral deficiency [14]. Furthermore, early ejaculation and mental disturbance were seen during the **counselling**. We previously observed a data gap for sperm samples from smokers and drinkers [15]. Cryo-preservation is used to preserve sperm cells for males in couples undergoing vasectomy and other therapies that influence their reproductive rate, such as chemotherapy and radiation therapy [16], [17]. In most cases, the medium used to preserve sperm contains glycerol, and sucrose is frequently added.

This approach can occasionally result in sperm DNA mutations. Furthermore, several studies use various media to preserve human and animal sperm cells (egg yolk, citrate, EDTA, zinc, NA, K, trehalose, penicillin, streptomycin, various plant materials, and polyphenols) [18]. However, sperm's shelf life was shortened and reported. As a workaround, an alternative approach of introducing nano emulsions to protect sperm cells was developed based on the dosage used. The current investigation of the small size of the droplets (nano emulsions) enables homogenous deposition on substrates. They are appropriate for the effective delivery of active substances [8]. They have been manufactured in various doses and do not harm healthy human and animal cells, making them even more appropriate for medicinal reasons. As a result, it is widely used in numerous sectors, particularly cancer therapy. It also boosts the absorption rate and eliminates absorption fluctuation. As a result, it has been chosen for infertility treatment by cryopreservation. The present study aims to determine the characteristics of sperm morphology, stability tests, and changes in several sperm parameters in fertile and infertile males. We discovered a link between stability tests and infertile sperm morphology.

Furthermore, research shows that a lower amount of stable sperm in the sperm can disrupt the function of the sperm, causing various alterations and infertility. Reduced fertility arises from reducing the number of stable spermatozoa in the sperm. The stability of the sperm is critical in the maturation of spermatozoa's fertilizing activity [19]. Some non-obstructive azoospermia infertile patients have zero or fewer steady spermatozoa activity, resulting in sperm membrane non-utilization. Assume that more stable sperm in sperm can result in a normal morphological condition in infertile seminal fluid [9 & 19]. This idea has yet to be validated in patients who use nano emulsions with sperm and can be linked to sperm fertility. Meanwhile, the sperm membrane performs numerous critical tasks in sperm stability and shape. However, we established a relationship between sperm morphology and sperm parameters in fertile and infertile individuals when using CN oil nano emulsions.

## 5 Conclusion

Cryo-preservation with nano emulsions of cinnamon oil (CN), clove oil (CE), and eucalyptus oil (EL) produces the best outcomes, as when the samples were cryopreserved using cinnamon oil (CN), they produced better morphology sustainability, viability and vitality than eucalyptus oil (EL) and clove oil (CL).

## CRediT authorship contribution statement

**Parameswari R:** Writing-review & editing, Writing-original draft, Validation, supervision, resources, Project administration, Methodology, Investigation, Formal analysis, Data curation, Conceptualization. **Sridharan TB:** Validation, supervision, Project administration, Conceptualization. **Babujanarthanam R:** Investigation, Data curation, Conceptualization. **Madhan Kumar P:** Visualization, Resources, Investigation, Formal analysis, Data curation. **Ganesamoorthy R:** Validation, Investigation. **Thirugnanasambandham K:** Visualization,Validation, Resources.

## Declaration of Competing Interest

The authors declare that they have no known competing financial interests or personal relationships that could have appeared to influence the work reported in this paper.

## Data Availability

The data that has been used is confidential.
